# A Case Study Report: Crisis Emergency Admission of an Autistic and Moderate ID (Intellectual Disability) Patient and an Overview of Multidisciplinary Therapeutic Interventions Provided by a General Adult Psychiatry Clinical Team That Significantly Reduced Risk Incidents and Improved Patient Outcomes

**DOI:** 10.1192/bjo.2025.10751

**Published:** 2025-06-20

**Authors:** Angela Misra, Omer Malik, Emma Hahn, Jeremy Stileman, Karin Dicander

**Affiliations:** Cygnet Healthcare, London, United Kingdom

## Abstract

**Aims:** An increasing number of patients with Autism Spectrum Disorder (ASD) and Intellectual Disability (ID) are being admitted to general psychiatric wards and managed by general psychiatrists. This case report describes a crisis admission and reviews the models of care, interventions, and outcomes delivered by a non-specialist multidisciplinary team (MDT) following the closure of a specialist ID unit.

**Methods:** X is a 30-year-old female with ASD and Moderate ID, presenting with complex self-harming behaviours (self-punching, head-banging), psychogenic polydipsia, self-neglect, and risks to others (aggression, property damage). She required 2:1 staffing observations. After the closure of the specialist ID ward, X was transferred to Cygnet Churchill Hospital in January 2024, initially for community discharge, but an unforeseen admission necessitated continued complex care.

**Results:** An interdisciplinary intervention programme including carer-informed change in antipsychotic medication and Clinical Genetics history review. Psychological interventions targeted three areas: patient-centred care, MDT-centred care, and personalized risk assessment. Patient-centred interventions involved exploring emotions and dysregulation management, with X identifying strategies to manage emotional regulation and self-expression. MDT interventions included rapid PBS training to upskill non-specialist staff in managing ID and ASD, alongside discontinuing communication aids. A personalized START risk assessment, integrating five case-specific items, enhanced X’s understanding of her behaviours.

The Vona du Toit Model of Creative Ability (VDTMoCA) was applied to create an individualized intervention plan promoting choice, including an interest checklist, healthier eating options, choice cards, and reformatted social stories tailored to X’s preferences. These strategies developed X’s Occupational identity and supported emotional regulation.

The comparison of pre- and post-admission Global Assessment of Progress (GAP) Scores showed a 44.7% improvement, with the highest gains in complex challenges (50%), Daily Living Skills Observation Scale (41.7%), Family Caregiver Support Program (64.2%), and Daily Risk Assessment (DRA). There was a significant reduction in self-harm (81.25%) and physical aggression (28.13%), but an increase in restraint (52.4%), verbal aggression (350%), property damage (183.3%), and absconding (33.3%). No changes were observed in rapid tranquillization or medication adherence. Discarding communication mats and Makaton led to notable improvements, with X independently chairing her ward rounds for the past four months, demonstrating progress in self-advocacy.

**Conclusion:** This case highlights the importance of regular reviews of long-stay patient interventions and demonstrates that general adult psychiatrists, when supported by interdisciplinary teams, can achieve significant improvements in managing complex cases, leading to better outcomes for individuals with ASD and ID.

